# Machine Learning Techniques Reveal Aberrated Multidimensional EEG Characteristics in Patients with Depression

**DOI:** 10.3390/brainsci13030384

**Published:** 2023-02-22

**Authors:** Gang Li, Hongyang Zhong, Jie Wang, Yixin Yang, Huayun Li, Sujie Wang, Yu Sun, Xuchen Qi

**Affiliations:** 1 Key Laboratory for Biomedical Engineering of Ministry of Education of China, Department of Biomedical Engineering, Zhejiang University, Hangzhou 310058, China; 2 College of Mathematical Medicine, Zhejiang Normal University, Jinhua 321004, China; 3 College of Mathematics and Computer Science, Zhejiang Normal University, Jinhua 321004, China; 4 College of Foreign Language, Zhejiang Normal University, Jinhua 321004, China; 5 College of Teacher Education, Zhejiang Normal University, Jinhua 321004, China; 6 Key Laboratory of Intelligent Education Technology and Application, Zhejiang Normal University, Jinhua 321004, China; 7 Department of Neurosurgery, Shaoxing People’s Hospital, Shaoxing 312000, China; 8 Department of Neurosurgery, Sir Run Run Shaw Hospital, Zhejiang University School of Medicine, Hangzhou 310016, China

**Keywords:** depression, electroencephalogram (EEG), machine learning, feature selection, functional connectivity, power spectral density, fuzzy entropy

## Abstract

Depression has become one of the most common mental illnesses, causing serious physical and mental harm. However, there remain unclear and uniform physiological indicators to support the diagnosis of clinical depression. This study aimed to use machine learning techniques to investigate the abnormal multidimensional EEG features in patients with depression. Resting-state EEG signals were recorded from 41 patients with depression and 34 healthy controls. Multiple dimensional characteristics were extracted, including power spectral density (PSD), fuzzy entropy (FE), and phase lag index (PLI). These three different dimensional characteristics with statistical differences between two groups were ranked by three machine learning algorithms. Then, the ranked characteristics were placed into the classifiers according to the importance of features to obtain the optimal feature subset with the highest classification accuracy. The results showed that the optimal feature subset contained 86 features with the highest classification accuracy of 98.54% ± 0.21%. According to the statistics of the optimal feature subset, PLI had the largest number of features among the three categories, and the number of beta features was bigger than other rhythms. Moreover, compared to the healthy controls, the PLI values in the depression group increased in theta and beta rhythms, but decreased in alpha1 and alpha2 rhythms. The PSD of theta and beta rhythms were significantly greater in depression group than that in healthy controls, and the FE of beta rhythm showed the same trend. These findings indicate that the distribution of abnormal multidimensional features is potentially useful for the diagnosis of depression and understanding of neural mechanisms.

## 1. Introduction

Depression is a neurological syndrome with three main symptoms, which refer to slow thinking, low mood, and cognitive impairment [1,2,3]. So far, depression has been one of the most common mental illnesses and has evolved into a global problem [4]. Most humans can be victims of depression, ranging from children to the elderly [5]. According to the statistics from the World Health Organization, there have been more than 350 million cases of depression in the world [6]. Moreover, approximately 54% of people who commit suicide each year were suffering from depression [7]. The detrimental effects of depression on families and society are enormous. However, due to the lack of uniform and accurate neural markers, it is difficult to objectively diagnose depression. Therefore, an accurate understanding of the neural mechanisms of depression is crucial and significant for the diagnosis of depression.

With the development of noninvasive neuroimaging techniques, electroencephalogram (EEG), functional magnetic resonance imaging (fMRI), and magnetoencephalography (MEG) have been used to capture information about brain activity [8,9,10]. Currently, these brain imaging techniques have been used to assist the diagnosis of various brain mental disorders [11,12,13]. Among them, EEG is widely applied to assess the abnormal brain wave changes caused by depression with surprising success due to its simplicity, affordability, and high temporal resolution [14,15]. A previous study pointed out that EEG is a nonlinear and non-smooth signal generated by complex brain systems and has multidimensional physiological information [16]. The traditional research method of EEG focused on analysis of linear features such as time–frequency domain [17]. The linear features of the temporal and frequency distributions can explicitly characterize the periodic activity of the brain [18,19]. Grin-Yatsenko et al. found that alpha and beta rhythms were significantly increased in patients with depression during the closed and open-eye rest states, indicating the reduced activation of the cerebral cortex in patients with depression [20]. Considering the properties of EEG signals, nonlinear features were also used in the study of EEG by virtue of their conformity to the qualities of EEG signals in patients with depression [21,22]. Chen et al. reported that the nonlinear features were more significantly different between the depression and control groups as well as sensitive to the analysis of frontal EEG signal complexity in depression [23]. Furthermore, many of the symptoms and deficits of depression are thought to be caused by dysfunction of brain networks connecting the limbic system and cortical areas [24,25]. Characteristics of functional connectivity that characterize the transfer of information between different brain regions are widely applied [26,27,28]. As an example, Park et al. showed that information transfer between different areas of the brain was severely disrupted in patients with depression using the synchronization likelihood measures [29]. With diverse characteristics that have been used to decode EEG signals, this has promoted a better understanding of abnormal neurological changes in patients with depression.

In recent decade, combined with machine learning algorithms, many EEG characteristics have performed an important position in the auxiliary diagnosis of depression [10]. Li et al. used an ensemble learning to process the linear feature power spectral density, generated new features, and obtained satisfactory accuracy for classification using support vector machine (SVM) [30]. In addition, previous EEG studies have shown that the pathogenesis of depression is related to abnormal functional connectivity between different regions of the brain [15,24]. In a recent study, Mumtaz et al. performed classification of depressed and healthy individuals, and found that the accuracy was as high as 98% when using SVM to classify functional connectivity features [31]. In addition, previous studies have used feature selection algorithms to remove redundant features to improve the recognition accuracy and reduce model complexity [32,33]. Linear and nonlinear features of the three channels (Fp1, Fp2, and Fz) were extracted, and the minimum redundancy maximum correlation feature selection method was used to identify depression with an accuracy of 79.19% [34]. Ghiasi et al. extracted the spectral and functional connectivity features of patients with depression and healthy controls and fed them into an SVM classifier embedded in the recursive feature elimination (RFE) algorithm, obtaining 83.91% classification accuracy [35]. Up to now, many studies have been presented using machine learning and EEG characteristics to identify depression.

In this study, we constructed a framework based on machine learning to analyze multidimensional EEG features to gain insight into the abnormal neural mechanisms prominent in patients with depression. Machine learning algorithms were used to pick out the multidimensional characteristics that were important for classification of depression. We recorded the EEG signals from the depression group and healthy controls with eyes closed in the resting state. Then, three different categories of characteristics (PSD, FE, and PLI) were used to extract EEG characteristics. PSD presents the power distribution per frequency and can reveal the variation pattern of complex hidden periodic signals in the frequency domain [36]. FE has a strong application to short-time sequences contaminated by noise, which is based on fuzzy theory to assess the complexity of the system [37]. PLI is used to estimate the asymmetry of the phase difference distribution of any two channel EEG signals [38]. These three methods decoded the information in EEG signals in three different dimensions (linear feature, nonlinear features, and functional connectivity feature), and they described the neurophysiological significance of EEG signals from three different perspectives. Then, the features with statistical differences between the patients with depression and healthy controls were picked out and then sequenced. According to the sorting order, the statistically different features were put into the machine learning classifiers to obtain the optimal subset of features with the highest classification accuracy. This study aims to reveal important neurobiological features of depression through the proposed analytical framework and achieve better classification performance for the diagnosis of depression.

## 2. Materials and Methods

### 2.1. Participants

Forty-one patients (10 males and 31 females) with depression were recruited by the local hospital to engage in this study. All of them were evaluated by professional psychiatrist interviews. The age of the patients with depression ranged from 19 to 61 years, and the mean age was 45.22 ± 11.80. All subjects complained of illness for more than 1 month. Thirty-four healthy controls (11 males and 23 females) were recruited from the local community. The age range of the healthy controls was between 21 and 57 years, and the mean age was 40.18 ± 11.67. There was no statistical difference in age between the depression group and the healthy controls.

All participants filled out a questionnaire of the Hamilton Depression Inventory 17-item scale (HAMD-17). The following requirements were met: HAMD-17 ≥17 for depression; HAMD-17 ≤7 for healthy controls [39]. The clinical characteristics of the patients with depression and healthy controls are given in Table 1. In addition, participants were right-handed and had normal or corrected-to-normal vision. Each participant was prohibited from drinking alcohol and taking psychotropic drugs for 8 h before EEG recording. The whole EEG acquisition process was in a quiet environment without any other electromagnetic interference. The experiment was permitted by the Ethics Committee of Huzhou Third Municipal Hospital. Written informed consent was obtained from all participants before the test.

### 2.2. EEG Data Acquisition and Preprocessing

A 16-channel EEG acquisition device (Nicolet EEG TS215605) was used to record EEG signals according to international standard 10–20 system. The channels were Fp1, Fp2, F7, F3, F4, F8, T7, C3, C4, T8, P7, P3, P4, P8, O1, and O2. Prior to the start of the experiment, the participants closed their eyes for 5 min to reduce other biological artefacts. The electrode cap was fixed on the head, and the electrodes were fixed with conductive paste. Once the EEG signals turned smooth, the recording started and lasted for 10 min. The sampling rate was 250 Hz, and all electrode impedances were controlled below 5 kΩ.

A set of widely recognized preprocessing procedures was used to perform the necessary preprocessing of the raw EEG signals. The raw EEG signals were downsampled to 125 Hz and filtered between 4 Hz and 30 Hz using a digital pass filter of fourth-order Butterworth band. Visual screening and DC correction (embedding in EEGLab 10.2.5.8b) were used to exclude blinks and other kind of artefacts. Physiological artefacts were the most serious noise in EEG; in order to obtain more pure and effective EEG data, the widely used artefact elimination tool, independent component analysis (ICA), was used to remove artefacts such as eye movements [40]. All components of ICAs were inspected visually and manually selected for rejection according to the ADJUST plugin in EEGLab. Next, 4 s of continuous EEG data with 50% overlap were singled out as an EEG sample, resulting in 8731 samples for the depression group and 6924 samples for the healthy control group. Subsequently, the EEG data involved in task conversion were decomposed into four standard bands by a digital pass filter of fourth-order Butterworth band: theta (4–8 Hz), alpha1 (8–10 Hz), alpha2 (10–13 Hz), and beta (13–30 Hz). The delta rhythm (0.5–4 Hz), which is mainly related to sleep [41,42], was filtered during preprocessing, because the artefacts in the raw EEG data were superimposed with delta rhythm, and the preprocessing process would have lost some information about delta rhythm.

### 2.3. Multidimensional EEG Characteristic Extraction

When recording the EEG signals of patients with depression in the hospital, we found that physicians directly observe whether the amplitude of the EEG signals was abnormal to aid in the diagnosis of depression. It was considered that the diagnostic process depended on the subjective judgment of the physician, and that there was a high possibility of misdiagnosis. Studies have proven the feasibility of using EEG characteristics to diagnose depression with promising results [30,31]. The more frequently used EEG characteristics can be divided into three categories (linear features, nonlinear features, and functional connectivity). In this study, we extracted three representative characteristics (PSD, FE, and PLI) of them and they were calculated separately for each sample.

#### 2.3.1. PSD Extraction

PSD is a measure of the mean square value of a random variable, which is the average power scale per unit frequency. Its advantage is the transformation of EEG waves with amplitude varying with time into a spectrum of EEG power varying with frequency, thus allowing visualization of the distribution and transformation of EEG rhythms [36,43].

In this study, 16 EEG channels × 4 rhythms of PSD features were calculated for each sample using the periodogram method. The *pwelch* function of MATLAB was used for the power spectrum estimation of the periodogram method, which is a modified method for estimating the PSD of the periodogram. It segments the signal by adding windows to find its PSD, and then implements the averaging. Specifically, for the given EEG signal $X\left(n\right)$, its frequency spectrum $X_{N}\left(f\right)$ can be estimated by fast Fourier transform. Then, the power spectrum $P_{x}\left(f\right)$ is obtained from the modulo square of the spectrum, as in Equation (1). The EEG power of each rhythm can be derived from Equation (2). $E\left(h\right)$ is the power value of the h rhythm, and $f_{h}$ and $f_{l}$ are the upper and lower frequency limits of the h rhythm, respectively.

(1) $$P_{x}\left(f\right)=\frac{1}{N}{\left|X_{N}\left(f\right)\right|}^{2}.$$

(2) $$E\left(h\right)=\frac{1}{f_{h}-f_{l}}\int_{f_{l}}^{f_{h}}P_{x}\left(f\right)df.$$

#### 2.3.2. FE Extraction

Entropy is an important numerical characteristic of fuzzy variables, which is used to measure the uncertainty of fuzzy variables. Fuzzy variables are variables that take values in uncertain fuzzy sets, and fuzzy entropy describes the degree of fuzziness of a fuzzy set. Fuzzy entropy uses a fuzzy function to reflect the similarity of samples and works well for signals other than nonlinear stationary [44,45].

For the given EEG signal of length N, $x\left(1\right)$, $x\left(2\right)$, …, $x\left(N\right)$, the m-dimensional vector is defined as in Equation (3). $u\left(i\right)$ is shown in Equation (4). The distance between two m-dimensional vectors $X_{m}\left(i\right)$ and $X_{m}\left(j\right)$ is defined by Equation (5). The similarity between vectors $X_{m}\left(i\right)$ and $X_{m}\left(j\right)$ is $A_{ij}^{m}$, which is calculated using Equation (6). Defining the function as in Equation (7), we can obtain Equation (8). The fuzzy entropy of the EEG signal is calculated using Equation (9), where m is the embedding dimension and r is the tolerance parameter.

(3) $$X_{m}\left(i\right)=\left\{x\left(i\right),x\left(i+1\right),\cdots x\left(i+m-1\right)\right\}-u\left(i\right)\;\left(i=1,\cdots ,N-m+1\right).$$

(4) $$u\left(i\right)=\frac{1}{m}\overset{m-1}{\underset{j=0}{\sum }}x\left(i+j\right).$$

(5) $$d_{ij}=\underset{k\epsilon \left(1,m-1\right)}{\max}\left\{\left|x\left(i+k\right)-u\left(i\right)-\left(x\left(j-k\right)-u\left(j\right)\right)\right|\right\}\;\left(j\neq i\right).$$

(6) $$A_{ij}^{m}=exp\left(-ln\left(2\right)\cdot {\left(\frac{d_{ij}}{r}\right)}^{2}\right).$$

(7) $$C_{i}^{m}\left(r\right)=\frac{1}{N-m}\overset{N-m+1}{\underset{j=1,j\neq i}{\sum }}A_{ij}^{m}.$$

(8) $$\emptyset^{m}\left(r\right)=\frac{1}{N-m+1}\overset{N-m+1}{\underset{i=1}{\sum }}C_{i}^{m}\left(r\right).$$

(9) $$FuzzyEn\left(m,r,N\right)=ln\emptyset^{m}\left(r\right)-ln\emptyset^{m+1}\left(r\right).$$

In the present study, a typical value for the embedding dimension m was set as 2, and the value r was determined by k×δ; N is the length of the EEG signal under observation (*N* = 4 s × 125 Hz = 500). The value of k was set to 0.2, which usually takes a range of 0.10 and 0.25 [46,47]; δ is the standard deviation of the EEG signal.

#### 2.3.3. PLI Extraction

PLI is an indicator to measure the degree of phase synchronization between two oscillating signals [38]. It can be estimated using Equation (10). N is the timepoint. sign is a symbolic function whose output is 1 when the independent variable is positive, −1 when the independent variable is negative, and 0 for 0. $\varphi_{rel}$ denotes the phase difference between the two channel signals at time $t_{n}$. The value of PLI ranges from 0 to 1, and a higher value indicates a stronger degree of phase synchronization. PLI is widely used in EEG studies by virtue of its low sensitivity to volume conduction [48,49]. In this study, PLI was used to calculate the phase synchronization index between any two electrodes, which resulted in 120 connectivity values (16 × (16 − 1)/2 functional connections × 4 rhythms) in each sample.

(10) $$PLI=\left|\left\{sign\left(\Delta \varphi_{rel}\left(t\right)\right)\right\}\right|=\left|\frac{1}{N}\overset{N}{\underset{n=1}{\sum }}sign\left(\Delta \varphi_{rel}\left(t_{n}\right)\right)\right|.$$

### 2.4. Features Ranking and Selection

Considering the relatively large number of features (i.e., PSD, 16 EEG channels × 4 rhythms; FE, 16 EEG channels × 4 rhythms; PLI, 16 × (16 − 1)/2 functional connections × 4 rhythms; 608 features in total) that may contain irrelevant and redundant features, a feature selection method was needed. In this study, one-way analysis of variance (ANOVA) was carried out to determine statistically significant differences in FE, PSD, and PLI characteristics between the depression group and healthy controls (*p* < 0.05). To remove features that may be considered redundant and minimize classification bias due to overfitting, the feature selection algorithms random forest (RF), mutual information (MI), and support vector machine recursive feature elimination (SVM-RFE) were used to isolate workload-related features and integrate them into a subset of optimal features, respectively. RF is a machine learning ensemble algorithm, which uses random resampling technology and random node splitting technology to construct multiple decision trees and obtains the final classification result through voting. Specifically, the RF algorithm determined the importance of each feature by calculating the amount of contribution of that feature in each decision tree. The contribution was calculated by resolving the Gini index before and after branching for the feature on a node. Then, the same process was used to obtain the contribution of other features. Finally, the average value of the change in the Gini index for one feature of all decision trees was calculated, and the importance of the features was ranked according to the magnitude of the value [50]. In this study, 500 decision trees were used in the RF algorithm. SVM-RFE is a sequential backward selection algorithm based on the maximum interval principle of SVM [51]. All features should be put into the SVM-RFE model for training to obtain weights, and the feature with the lowest weights is removed. Then, the model is trained again using the remaining features, and this step is iterated until there are no features. The kernel function used in SVM was the radial basis function (RBF) kernel. Mutual information (MI) is a measure of statistical independence that has two properties [52]. Firstly, it can measure any kind of relationship between random variables, including nonlinear relationships. Secondly, MI is invariant under the transformations of the feature space, such as translation, rotation, and any transformation that preserves the order of the original elements of the feature vector.

The result of each feature ranking was independent, and the position of the ranking determined its importance. In order to get stable and reliable ranking result, 100 iterations of the RF feature ranking process were performed. The result was a matrix of 100 × n feature ranking sets (n denotes the statistically significant features in each rhythm). The most common feature in the first column from 100 × n was selected as the first feature, the two most common features in the first and second columns were selected as the first and second features, etc., following this rule to generate the final feature ranking result.

**Figure 1:**
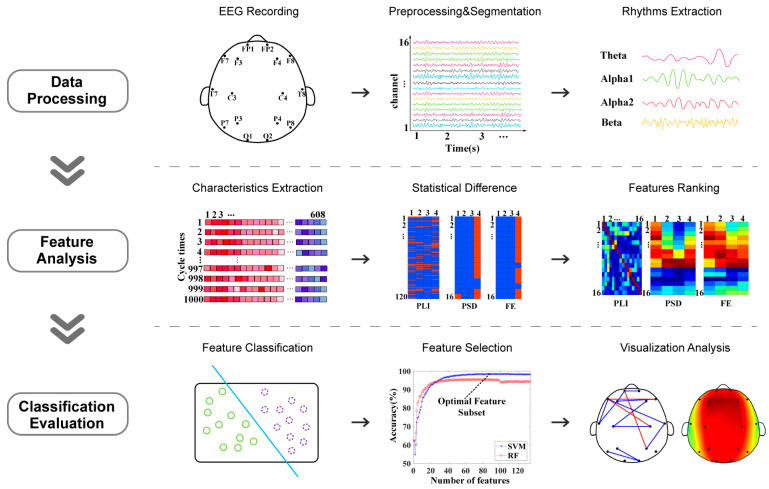
Schematic flowchart of data analysis.

Upon completion of the above work, the optimal subset of features to distinguish patients with depression from healthy individuals was investigated by performing the classification task with a single feature as the additional amount at each time according to the position of the feature ranking. This step was repeated until all features were added to the classifiers. SVM and RF were used to distinguish depression group and healthy control group; the SVM used a radial basis function (RBF) kernel function, and the RF used 500 decision trees. The holdout method was used to divide the dataset applied in the classification, with 80% of the samples from each of the depression and healthy controls selected as the training set and the remainder as the test set. A schematic flowchart of the data analysis method of this study is shown in Figure 1. All analyses, including EEG preprocessing, feature calculations, statistics, and classifications, were implemented using MATLAB 2019b (Mathworks Inc., Natick, MA, USA).

## 3. Results

The different feature selection models with highest accuracy are shown in Table 2. It was found that, when using SVM as the classifier and RF as the feature selection model, the highest accuracy of the optimal feature subset was up to 98.54% ± 0.21%, which is higher than the 95.52% when using RF as the classifier. The optimal subset of features selected from the 134 statistically features contained 86 features. Table 3 demonstrates the distribution of the features contained in the optimal feature subset. It can be seen that there were 59 PLI features (8/59 for theta, 14/59 for alpha1, 9/59 for alpha2, and 28/59 for beta). The overall number of features of the PSD was 15 (1/15 for theta and 14/15 for beta). The number of FE features presented in the optimal feature subset was 12, all of which were concentrated in the beta rhythm.

**Figure 2:**
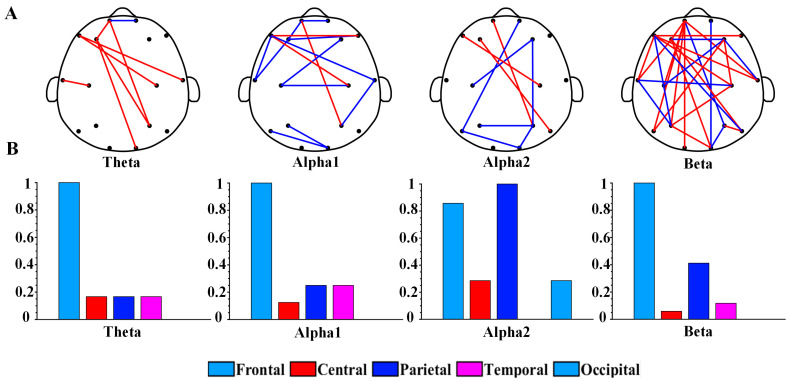
(**A**) Brain network topology of PLI functional connections of theta, alpha1, alpha2, and beta rhythms in optimal feature subset. The blue edges represent that the PLI values of the depression group are smaller than that of the control group, while the red edges represent that the depression group is greater than the control group. (**B**) Values after normalization of the number of functional connections in different brain regions for each rhythm.

Figure 2 shows the topography of the brain network and the distribution of brain regions of PLI in the optimal feature subset. To compare the connected values between the depression group and the healthy controls, we averaged the PLI matrices for all samples of the two groups of participants separately. Figure 2A shows the results of the topography functional connectivity analysis. Specifically, 88% of functional connections in the theta rhythm were greater in the depression group than in the healthy controls. Additionally, 64% of functional connections in the beta rhythm were greater in the depression group. Moreover, 79% of functional connections in the alpha1 rhythm and 78% of functional connections in the alpha2 rhythm had lower values in the depression group than in the healthy controls. Figure 2B shows the topological distribution of PLI functional connections in each brain region. The functional connections of theta, alpha1, alpha2, and beta rhythms were mainly distributed within the frontal region (Fp1, Fp2, F3, F4, F7, and F8) and other brain regions. Overall, the proportion of functional connectivity associated with frontal regions was 76% (7/8 for theta, 10/14 for alpha1, 6/9 for alpha2, and 22/28 for beta).

**Figure 3:**
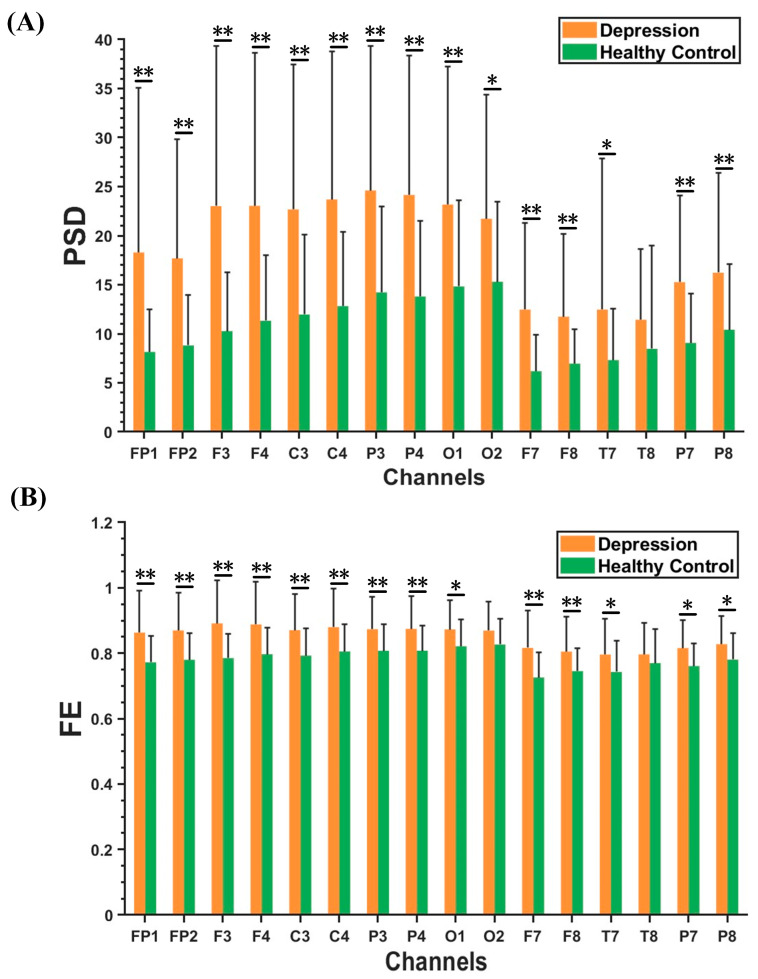
Results of PSD and FE features in beta rhythm between depression group and healthy controls. (**A**) PSD results. (**B**) FE results. The results are given as the mean + standard deviation; * *p* < 0.05, ** *p* < 0.01.

Figure 3A**,**B demonstrate the results of beta rhythm in PSD and FE characteristics within the optimal feature subset between the depression group and healthy controls. Compared to the healthy controls, FE and PSD had higher values in the depression group. Moreover, the PSD characteristics of beta rhythm in the depression group had significantly greater values in the central, parietal, and occipital regions than in other brain regions. The FE characteristics of the beta rhythm in the depression group showed much larger values in the frontal, central, and parietal regions.

## 4. Discussion

In this study, an analytical framework based on machine learning and multidimensional EEG features was used to highlight abnormal brain activity in patients with depression. The significant findings are as follows: firstly, the optimal feature subset contained 86 features selected from 134 statistically significant features, with the highest classification accuracy of 98.54% ± 0.21% between the depression group and healthy controls. Secondly, the number of PLI features in the optimal feature subset was significantly larger than that of PSD and FE. Moreover, compared with other rhythms, the characteristics of the beta rhythm had the highest proportion. Thirdly, compared to the healthy group, the PLI values of the theta and beta rhythms in the optimal feature subset were increased, while those of the alpha1 and alpha2 rhythms were decreased in the depression group. The PSD results of the theta and beta rhythms were significantly greater in depression group than in the healthy controls, and the FE of the beta rhythm showed the same trend.

### 4.1. Machine Learning Effectively Extracted EEG Features

Until now, several studies have shown that EEG characteristics are widely used to diagnose depression [53,54]. Different kinds of features can decode EEG signals from various perspectives, but not all features are correlated with the diagnosis. In this study, machine learning methods were used to extract effective features and remove redundant features, resulting in an optimal feature subset that contained 86 features and obtained the highest classification accuracy of 98.54% ± 0.21%. In a previous study, Li et al. used the integration model and obtained 89.02% accuracy in a study of depression recognition, and they found significant differences between depressed and normal individuals in the temporal lobe [30]. Hosseinifard et al. obtained 90% accuracy of depressed and normal individuals by using four nonlinear features and a logistic regression classifier in their study of classification [54]. In addition, Zhang et al. used network features such as shortest path length and clustering coefficient to recognize depression with an accuracy of 93.3%. They noted that patients with depression showed a stochastic trend in the functional brain network and a weakening of small-world characteristics [55]. All of these aforementioned studies demonstrated that machine learning methods can effectively recognize depression with EEG data. The high recognition accuracy obtained in this study demonstrated that multidimensional EEG characteristics and machine learning methods of feature selection algorithms improved the recognition rate of depression, thereby providing new insight into the abnormal brain neurological conditions of patients with depression. The highest proportion of PLI in the optimal feature subset indicated that functional connectivity could successfully characterize abnormal brain activity in patients with depression. Previous research has shown that patients with depression suffer from a weakened regional function and abnormal communication in different brain regions [56,57]. In addition, the present study corroborated the importance of PLI in identifying depression compared to PSD and FE. Similarly, Sun et al. found that PLI features were superior to linear features and nonlinear features when using different types of features to identify depression [49]. Leuchter et al. demonstrated significant differences in functional brain connectivity patterns between subjects suffering from depression and healthy controls [58]. Varone et al. found that functional connectivity analysis may be more effective than PSD in identifying psychogenic nonepileptic seizures within scalp EEG time series in the PNES study [59]. In summary, our work further supports the importance of functional connectivity in the parsing of EEG in depression from the perspective of multidimensional features.

In the present study, it was found that the FE and PSD features of the beta rhythm had the highest proportion in the subset of optimal features. Compared to other rhythms, the abnormal performance of the beta rhythm reflects the participant’s inattention and emotional abnormalities [60,61]. Previous studies also reported significant differences in FE and PSD of beta rhythm between the depression and control groups, which are consistent with our findings [53,62]. In this study, the machine learning methods effectively extracted EEG features and demonstrated which types of features played a more important role in the division of depression and healthy controls.

### 4.2. Alterations Occurring in Frontal Functional Connections

The PLI feature used in this study is a quantity for assessing the degree of phase synchronization between any two EEG channels [38,48]. In this study, the theta, alpha1, alpha2, and beta rhythms showed similar performance in that the PLI connections in the optimal feature subset were mainly distributed within frontal regions and other brain regions. The PLI values of the theta and beta rhythms increased in the depression group, while those of the alpha1 and alpha2 rhythms decreased. We can conclude that functional connectivity was disturbed in patients with depression.

The significantly altered functional brain connectivity in patients with depression is mainly associated with frontal areas. It is well known that the frontal lobe is mainly related to motor function, cognitive function, and mental activity [63,64,65]. A previous study found that frontal lobe dysfunction is a core symptom of depression [66]. Moreover, structural imaging studies demonstrated that patients with depression have a reduced volume of the prefrontal cortex, which demonstrates that depression causes substantial changes in the brain [67]. Olbrich et al. also showed that patients with depression are characterized by altered functional brain connectivity in frontal regions [57]. These studies could justify the findings of this research. In addition, some studies suggested that frontal asymmetry may be a neural marker of depression risk [63,64,68]. In this study, PLI functional connectivity features undergo multiple rounds of screening by machine learning algorithms, and the reduction in the number of PLI prompted a nonsignificant functional connectivity asymmetry in the prefrontal region. The findings indicated that significantly dysregulated functional connections in patients with depression are mainly distributed between the frontal area and other brain areas.

Compared to healthy controls, the phase synchronization of EEG signals is significantly altered in patients with depression, with the depression group showing increased PLI values for theta and beta rhythms and decreased values for alpha1 and alpha2. In a study of depression with different severities, Mohammadi et al. indicated that PLI values of the alpha rhythm in the depression group were significantly smaller than in the control group, which is consistent with our findings [69]. Kalev et al. found that the PLI of the beta rhythm was significantly higher in the depression group [70], which provides support for our findings to some extent. Liu et al. recorded the EEG signals during music perception in patients with depression and controls and indicated that patients with depression showed higher connectivity in the PLI of the beta rhythm [48]. These studies mentioned above could demonstrate the advantage of the beta rhythm in identifying depression. In addition, Zhang et al. found significantly higher synchronization of the theta rhythm in the frontal, central, and left temporal lobes in depression [55]. However, compared to the increase of PLI in beta and theta rhythms, few studies noted the decrease in PLI values in alpha rhythms among patients with depression, which could be a new finding. With the in-depth study of brain functional connectivity in depression, increasing evidence demonstrates that functional connectivity in the frontal lobes is significantly altered in patients with depression.

### 4.3. Significantly Altered Beta Rhythm

This study showed that the beta rhythm features of PLI, FE, and PSD had the highest number in the optimal feature subset (47% for PLI, 93% for PSD, and 100% for FE). The beta rhythm is generally associated with the alertness and arousal states of the brain, as recognized by many researchers [60,71]. To date, studies have shown that the functional connectivity of the beta rhythm in patients with depression is significantly different from that in healthy people [53,58]. Furthermore, abnormal manifestations of the beta rhythm have also attracted attention in other studies of psychiatric disorders based on EEG [72,73]. The high proportion of beta rhythms in multidimensional features provides fundamental theoretical support for the selection of neural markers for the future diagnosis of depression.

Compared to healthy controls, patients with depression showed a significant increase in the PSD and FE characteristics of the beta rhythm. Some studies have demonstrated that patients with depression exhibit a significant increase in beta rhythm spectrum power [20,53]. Moreover, Yang et al. found that the sample entropy of patients with depression was greater than that of healthy controls [74]. In the present study, we found that the characteristics of beta rhythm play a vital part in identifying depression. The increase in PSD and FE of the beta rhythm indicates that the brain activity of depressed patients is in a state of tension and neurological disorder. As an explanation for the appearance of abnormal brain activity in patients with depression, a study suggested the phenomenon of an increase in negative brain activity in specific EEG rhythms [75]. Another study indicated that it is an overreaction of the brain in maintaining a homeostatic state [76]. In addition, Grin-Yatsenko et al. reported a significant increase in the spectral power of the beta rhythm in patients with depression, which could potentially be associated with anxiety, and anxiety symptoms are likely to play an important role in the development of depression [20]. In conclusion, the significant changes in the beta rhythm of FE, PSD, and PLI features reveal the neural mechanisms of depression. This may be used as a neural marker in the future diagnosis of depression.

### 4.4. Limitations

Some limitations should be considered when interpreting the results of this study. Firstly, the sample size and the EEG channel number were relatively small, and a larger sample size and high-density EEG data are needed to draw definitive conclusions. Secondly, there were gender and age imbalances in the subjects due to the failure to recruit enough subjects. Thirdly, the female menstrual cycle was not considered as an influencing factor when collecting the subjects. Lastly, we used the fixed EEG bands, as each particular individual has their own frequency band definition. We will explore the impacts of using individualized EEG bands on machine learning. In future studies, we will improve data collection conditions and concentrate more on controlling the influencing factors such as the age, gender, and menstrual cycle of the subjects.

## 5. Conclusions

In the present study, we constructed a multidimensional feature extraction and selection framework to investigate significant neural mechanisms in patients with depression. Machine learning methods were used to identify significantly important multidimensional EEG features in depression and controls. The results showed that the optimal feature subset contained 86 statistically different features with a classification accuracy of 98.54% ± 0.21%. In the optimal feature subset, the PSD values of the theta and beta rhythms were significantly larger in patients with depression, and the FE of the beta rhythm showed the same trend. Moreover, functional connections were mainly distributed within the frontal lobe and other brain regions. The depression group had increased PLI values for theta and beta rhythms and decreased values for alpha1 and alpha2. This study revealed the neural mechanisms of depression from the perspective of multidimensional features using machine learning and provided a possible beta rhythm biomarker for the diagnostic study of depression.

## Figures and Tables

**Table 1:** Clinical characteristics of the patients with depression and healthy controls.

Characteristics	Depression	Healthy Control	*p*-Value
Number	41	34	-
Gender: male/female	10/31	11/23	-
Age (years)	45.22 ± 11.80	40.18 ± 11.67	0.07
HAMD-17	24.39 ± 7.01	3.85 ± 1.35	3.86 × 10^−28^

**Table 2:** Classification accuracy under different feature selection models.

Feature Selection Models	Classification Models	Accuracy
MI	SVM	98.36% ± 0.24%
	RF	95.40% ± 0.44%
RF	SVM	98.54% ± 0.21%
	RF	95.52% ± 0.56%
SVM-RFE	SVM	98.29% ± 0.34%
	RF	95.32% ± 0.30%

**Table 3:** Number of features for each rhythm in the multidimensional features within the optimal feature subset.

Rhythms	PLI	PSD	FE
Theta	8	1	0
Alpha1	14	0	0
Alpha2	9	0	0
Beta	28	14	12

## Data Availability

Not applicable.
